# Analysis of the Enhanced Stability of R(+)-Alpha Lipoic Acid by the Complex Formation with Cyclodextrins

**DOI:** 10.3390/ijms14023639

**Published:** 2013-02-07

**Authors:** Naoko Ikuta, Hironori Sugiyama, Hiroshi Shimosegawa, Rie Nakane, Yoshiyuki Ishida, Yukiko Uekaji, Daisuke Nakata, Kathrin Pallauf, Gerald Rimbach, Keiji Terao, Seiichi Matsugo

**Affiliations:** 1 School of Natural Systems, College of Science and Engineering, Kanazawa University, Kakuma-machi, Kanazawa 920-1192, Japan; E-Mails: naoko.ikuta@cyclochem.com (N.I.); hiro-sugiyama@t.kanazawa-u.ac.jp (H.S.); kanazawa.bio@yahoo.co.jp (H.S.); r_ie20_v_x@yahoo.co.jp (R.N.); 2 CycloChem Bio Co., Ltd., KIBC654R 5-5-2 Minatojima-minamimachi Chuo-ku, Kobe 650-0047, Japan; E-Mails: yoshiyuki.ishida@cyclochem.com (Y.I.); yukiko.uekaji@cyclochem.com (Y.U.); daisuke.nakata@cyclochem.com (D.N.); keiji.terao@cyclochem.com (K.T.); 3 Institute of Human Nutrition and Food Science, Christian Albrechts University, Kiel D-24118, Germany; E-Mails: pallauf@foodsci.uni-kiel.de (K.P.); rimbach@foodsci.uni-kiel.de (G.R.)

**Keywords:** R(+)-alpha lipoic acid, cyclodextrins, particle distribution, enhanced stability, XRD analysis, complex formation

## Abstract

R(+)-alpha lipoic acid (RALA) is one of the cofactors for mitochondrial enzymes and, therefore, plays a central role in energy metabolism. RALA is unstable when exposed to low pH or heat, and therefore, it is difficult to use enantiopure RALA as a pharma- and nutra-ceutical. In this study, we have aimed to stabilize RALA through complex formation with cyclodextrins (CDs). α-CD, β-CD and γ-CD were used for the formation of these RALA-CD complexes. We confirmed the complex formation using differential scanning calorimetry and showed by using HPLC analysis that complexed RALA is more stable than free RALA when subjected to humidity and high temperature or acidic pH conditions. Scanning electron microscopy studies showed that the particle size and shape differed depending on the cyclodextrin used for complexation. Further, the complexes of CD and RALA showed a different particle size distribution pattern compared with that of CD itself or that of the physical mixture of RALA and CD.

## 1. Introduction

R(+)-alpha lipoic acid (1,2-dithiolane-3-pentanoic acid; RALA) was first isolated and chemically identified in 1951 by Lester Reed and colleagues. Alpha-lipoic acid (ALA) has a chiral center at its C6 carbon, leading to two enantiomers, R(+)- and S(−)-ALA, of which RALA is the naturally occurring compound.

RALA functions as a cofactor for mitochondrial enzymes, such as the pyruvate dehydrogenase and the alpha-keto-glutarate dehydrogenase, as well as the branched-chain alpha-keto acid dehydrogenase complex and, thus, plays a critical role in glucose and energy metabolism [1,2].

Further, RALA and its dihydro-form, which is produced via metabolic reduction, are powerful antioxidants, because of their properties as radical scavengers and their synergistic interaction with other antioxidants. Dihydro-RALA reduces glutathione, which is an important antioxidant for many physiological processes, plays an essential role in detoxification of xenobiotics and is needed for signal transduction. As an amphiphilic molecule, RALA has a partition coefficient of approximately 4/1 (o/w) [3], meaning that RALA, unlike other antioxidants, is soluble in both aqueous and non-aqueous media. These qualities make RALA a central player in the antioxidant network, which is why ALA is used as a treatment for age-associated diseases, such as diabetes and neurodegenerative diseases [4–6]. Nowadays, ALA is widely used as an anti-ageing compound in cosmetics and also as a food supplement.

Although it is possible to separate the ALA enantiomers, the bioactive RALA and S(−)-alpha lipoic acid (SALA), the commercially available ALA is the racemate. This is due to the fact that even though the pure enantiomer RALA is the bioactive form, it is unstable when exposed to low pH, light or heat [7].

RALA decomposes gradually at room temperature and easily polymerizes at temperatures higher than its melting point, which is at 46–49 °C. Therefore, the stabilization of RALA is of great interest for its industrial use, and several studies aiming to stabilize RALA have been carried out. Amongst these attempts to stabilize the molecule were, for example, the complex formation or encapsulation with chitosan [8,9]. However, the encapsulation efficiency of chitosan with RALA was limited in these studies. In the United States, RALA sodium salt (NaRALA) is sold as “stabilized RALA”. Although this salt is somewhat more stable than the acid [10], its stability remains rather poor, as has been demonstrated in our studies.

In order to stabilize RALA by complex formation, we used cyclodextrins (CDs). These cyclic oligosaccharides consist of six (α-CD), seven (β-CD) or eight (γ-CD) α-1,4-linked glucopyranose units, with a hydrophilic hydroxyl group on their outer surface and a hydrophobic cavity in their center. Since the linking bond between the glucopyranose units cannot rotate freely, CDs are not perfectly cylindrical molecules, but toroidal or cone shaped. Based on this structure, the primary hydroxyl groups are located on the narrow side of the torus, while the secondary hydroxyl groups are located on the wider edge. α-CD is widely used as dietary fiber, because it is not enzymatically digested and, thus, has no nutritional value. Contrarily, γ-CD is broken down into monosaccharides and, therefore, functions as an energy source.

CDs are capable of forming complexes with a variety of ionic and lipophilic substances by taking the entire molecule or part of it into their cavity. Such a molecular complex formation affects many of the physicochemical properties of the guest molecules, such as their aqueous solubility, stability or bioavailability. There are two reports on how the physicochemical properties or characteristics of the lipophilic guest molecules, such as the coenzyme Q10, curcumin, astaxanthin and docosahexaenoic acid, changed when using γ-CD as a host molecule [11,12].

Cyclodextrins are widely and successfully used in the food and drug industry to stabilize and improve physical properties of many compounds. There is one study on the physicochemical properties of alpha lipoic acid-cyclodextrin complexes showing that complex formation with cyclodextrin improves ALA solubility and stability towards heat exposure [13]. However, in this and other publications on lipoic acid complex formation with compounds, such as chitosan [9], the ALA racemate was analyzed, while in our study, we were able to focus on the bioactive enantiomer RALA. By using a chiral column, it was possible to separate RALA and SALA from the racemate.

In this study, we made RALA-CD complexes in order to stabilize RALA and examined their formation, shape and stability under heated conditions using differential scanning calorimetry, high pressure liquid chromatography and scanning electron microscopy. Furthermore, focusing on the use of the RALA-CD complexes for oral consumption, we imitated the stomach environment and evaluated the stability of the different complexes under acidic conditions.

## 2. Results and Discussion

### 2.1. DSC of RALA-CD Complexes

Complex formation of RALA and the different CDs can be evaluated using DSC, since the melting, boiling or sublimation points of these complexes usually shift to a different temperature compared to the melting or boiling point of free lipoic acid.

**Figure 1:**
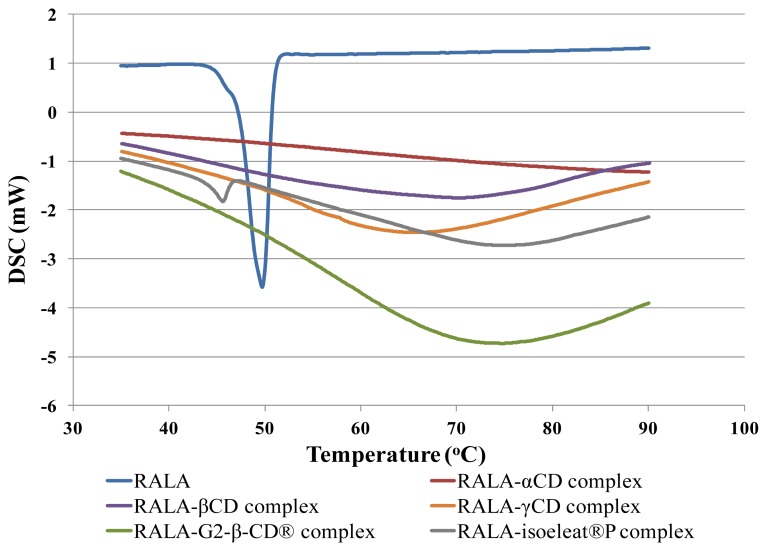
Differential scanning calorimetry (DSC) scans of R(+)-alpha lipoic acid (RALA) and RALA-cyclodextrin (CD) complexes. Samples were placed under the air flow for scanning and heating from 30 °C to 100 °C at 3 °C/min.

The DSC thermograms of RALA and the RALA-CD complexes are presented in Figure 1. As can be seen in this figure, RALA shows a characteristic endothermic fusion peak around 50 °C, while RALA-αCD, RALA-βCD, RALA-γCD and RALA-G2-β-CD^®^ did not show any clear peaks within the analyzed temperature range from 30 °C to 100 °C. However, the RALA-isoeleat^®^P complex showed a peak at 45.6 °C, indicating that isoeleat^®^P could not stabilize RALA. We calculated the uncomplexed RALA from the peak area ratio (RALA-isoeleat^®^P/RALA), and it was found that *ca*. 10% of the RALA contained in RALA-isoeleat^®^P was not stabilized. Based upon these results, it was deduced that complex formation of RALA with α-CD, β-CD, γ-CD or G2-β-CD^®^ had occurred and that the complexes led to an improved stability of their host molecule RALA.

Similar results were found in a previous study [13] in which racemic ALA complex formation with G2-β-CD^®^ and β-CD led to the disappearance of the endothermic DSC peak. Interestingly, ALA complex formation with α-CD, γ-CD and isoeleat^®^P by Mikuni and colleagues led only to a reduction of the peak sizes in the DSC thermogram. Since in our study the RALA-α-CD and RALA-γ-CD complexes did not exhibit a peak in the DSC scans, it appears that we have produced more stable RALA-CD complexes than Mikuni and colleagues. Regarding the complex formation with isoeleat^®^P, it seems that this RALA complex differed from the other RALA-CD complexes, as RALA-isoeleat^®^P shows a DSC peak at an even lower temperature than free RALA. This result indicates that decomposition or polymerization of RALA in the isoeleat^®^P-complex is likely to happen at even lower temperatures than would happen without complex formation. Therefore, it seems that this type of complex does not favor the stability of RALA.

A DSC scan of a polymerized RALA sample showed no peak within the analyzed temperature range (data not shown).

### 2.2. HPLC Analysis of the RALA Content in the RALA-CD Complexes

**Figure 2:**
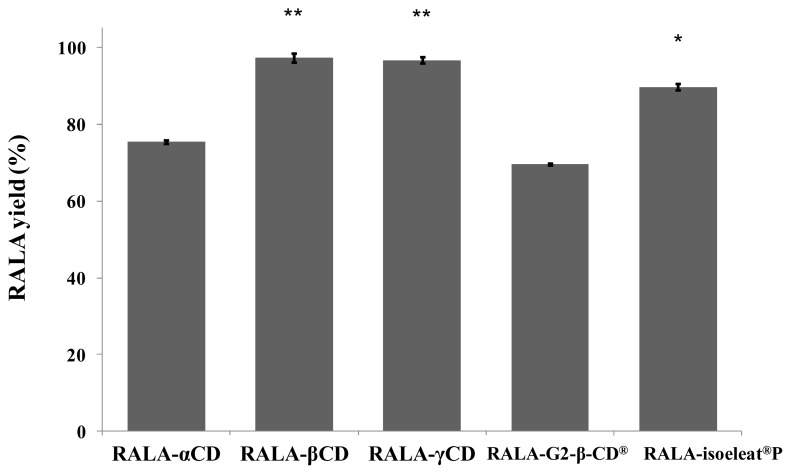
RALA yield after complex formation with the tested cyclodextrins (HPLC analysis). Yields were obtained based on the percentage of the measured RALA amount in the different RALA-CD complexes compared to the RALA amount added for complex formation. RALA-βCD and RALA-γCD yields were significantly higher than the other yields. ^**^p < 0.01 *vs.* RALA-αCD, RALA-G2-β-CD^®^ and RALA-isoeleat^®^P. ^*^p < 0.01 *vs.* RALA-αCD and RALA-G2-β-CD^®^ (Fisher’s least significant difference test).

The amount of RALA in the different RALA-CD complex preparations was measured using HPLC, and the results are shown in Figure 2. The yields are expressed as the percentages of RALA found in the complexes when compared to the amount of RALA used for the preparation of these complexes.

In the case of RALA-βCD and RALA-γCD, the yield was almost 90%, whereas the RALA-αCD and RALA-G2-β-CD^®^ complexes had a significantly smaller RALA yield of around 75% (Figure 2).

As mentioned in section 2.1, the RALA polymer shows no peak in the DSC scan, and therefore, polymerized RALA that could have been generated in the manufacturing process of the complex cannot be detected by the analytical methods used. However, it is likely that the missing RALA has been polymerized during complex generation.

Our results show that β-CD and γ-CD are more suitable for preparing RALA complexes than α-CD, G2-β-CD^®^ and isoeleat^®^P and that only a small amount of RALA is lost during the preparation of these complexes. There was a statistically significant difference between β-CD or γ-CD and the other CD complexes. Isoeleat^®^P also showed a significantly higher RALA yield than α-CD and G2-β-CD^®^. However, complex formation with isoeleat^®^P, α-CD or G2-β-CD^®^ is less efficient than β-CD and γ-CD, and a substantial amount of RALA seems to get lost in the manufacturing process of these complexes. This could be caused by RALA polymerization during complex preparation or by other factors that hinder a complete and stable complex formation of RALA with α-CD, G2-β-CD^®^ or isoeleat^®^P.

In these experiments, we have shown that the percentage of lost RALA raw material during the process of complex formation is smallest in the case of RALA-βCD and RALA-γCD.

### 2.3. Thermal Stability of RALA-CD Complexes

As mentioned in the introduction, RALA is unstable and easily polymerized when heated or exposed to light [7].

In this thermal stability test, RALA, NaRALA and RALA-CD complexes were exposed to 100% relative humidity for 30 min, 1 h, 2 h, 5 h, 24 h or 48 h at 25 °C, which is below the melting point of RALA, or 70 °C, which is above the melting point of RALA. After incubation, the remaining RALA was measured by HPLC.

**Figure 3:**
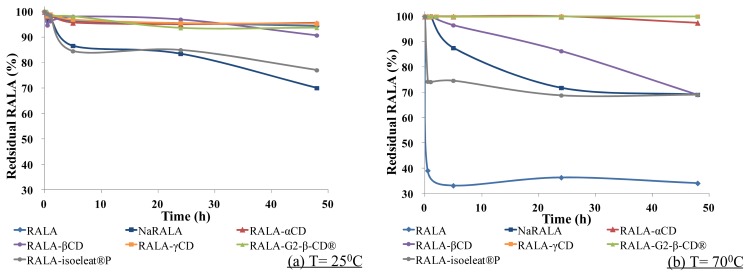
Stability of RALA complexes towards high temperatures. The samples were incubated at (**a**) 25 °C or (**b**) 70 °C and 100% relative humidity for 30 min, 1 h, 2 h, 5 h, 24 h and 48 h. The remaining amount of RALA was quantified using HPLC.

As shown in Figure 3a, at 25 °C (<melting point), free RALA was stable even under the humidity conditions. However, NaRALA, which is called “stabilized RALA” in the U.S., decomposed faster than free RALA, and the residual NaRALA was ca. 70% after 48 h.

The RALA-isoeleat^®^P complex was only slightly more stable than NaRALA, but the other complexes, RALA-αCD, RALA-βCD, RALA-γCD and RALA-G2-β-CD^®^, stabilized RALA, and more than 95% of the RALA remained in these complexes after 48 h at 25 °C/100% relative humidity.

As shown in Figure 3b, at 70 °C (>melting point)/100% relative humidity for 5 h, almost 70% of the free RALA had decomposed. Although NaRALA was more stable than free RALA under these conditions, a 13% loss of NaRALA was observed after 5 h and a 30% loss after 24 h. While the complex formation with α-CD, γ-CD and G2-β-CD^®^ improved RALA stability greatly with the recovery rates ranging from 97% (RALA-αCD) to almost 100% (RALA-γCD and RALA-G2-β-CD^®^) after 48 h, β-CD also improved RALA stability, albeit to a lesser extent. After 5 h, the RALA recovery was 96% for the RALA-β-CD complex, but the residual RALA decreased gradually over time, and after 48 h, barely 70% of the RALA was recovered (Figure 3b). However, although the residual percentage of RALA was higher after complex formation with isoeleat^®^P than in the non-complexed condition, the isoeleat^®^P complex does not withstand heat and humidity any better than the sodium salt. Over 5 h, the residual RALA in the RALA-αCD, RALA-γCD and RALA-G2-β-CD^®^ complexes did not change. However, the amount of remaining RALA in the RALA-βCD and RALA-isoeleat^®^P complexes decreased.

A possible explanation for the lower amount of remaining RALA in the RALA-isoeleat^®^P complex could be that this complex is less thermostable than the other cyclodextrin complexes. In the DSC (see Section 2.1), RALA-isoeleat^®^P was the only complex that showed a peak within the analyzed temperature range, indicating instability towards heat. In turn, the complexes without a peak in their DSC scans (RALA-αCD, RALA-βCD, RALA-γCD and RALA-G2-β-CD^®^) ameliorate the RALA yield after heat and humidity exposure, also when compared to NaRALA.

These results suggest that complex formation of RALA with the cyclodextrins α-CD, β-CD, γ-CD and G2-β-CD^®^ improves its thermal stability and its stability towards humidity.

These findings are in line with prior reports on improving racemic ALA stability regarding heat exposure through complex formation with cyclodextrins [13]. However, we were able to specifically analyze the remaining enantiomer RALA after exposure to high temperatures and have intensified the destabilizing treatment by incubating at 100% relative humidity.

Polymerization of RALA occurs via the S–S bond in the 1,2-dithiolane ring [7], and it is possible that the complex formation of RALA by cyclodextrins protects this S–S bond, thereby making the acid more stable.

Due to the poor stability of enantiopure RALA, the racemate containing SALA is sold, although RALA is the bioactive lipoic acid. Attempts to stabilize commercially available RALA have been fairly successful, as in the case of NaRALA, which is somewhat more stable than free RALA [10], although we have shown that complex formation with CDs, in terms of stability, is superior to the salt formulation.

As there seems to be a method to stabilize enantiopure RALA, further studies investigating the bioavailability of pure RALA in comparison to the racemic ALA and SALA would be possible. This is of especially high interest, as it is known that other racemic bioactive compounds, as for example vitamin E, differ in their kinetic, toxicological and biological properties [14]. It has been demonstrated that the naturally occurring RRR-α-tocopherol has a higher biopotency than the synthetically produced all-rac tocopherol and that the other α-tocopherol stereoisomers are bioactive, albeit to a lower extent than RRR-α-tocopherol [15,16]. To our knowledge, there is no data on the different RALA/SALA bioavailabilities and bioactivities. However, considering the widespread use of α-lipoic acid as a drug and food supplement [4], it seems very important to gain such knowledge and carry out relevant studies.

### 2.4. Stability of RALA-CD Complexes towards Low pH

Apart from studying the stability of the RALA-CD complexes towards heat exposure, where α-CD, β-CD, γ-CD and G2-β-CD^®^ provided better protection against RALA degradation than isoeleat^®^P, we additionally analyzed their stability under acidic conditions. Similar to heat and humidity, low pH also favors RALA polymerization, therefore leading to poor absorption in the stomach. In order to imitate the acidic environment in the stomach, we exposed free RALA and the RALA-CD complexes to pH 1.2 and incubated the samples at 37 °C for 1 h.

**Figure 4:**
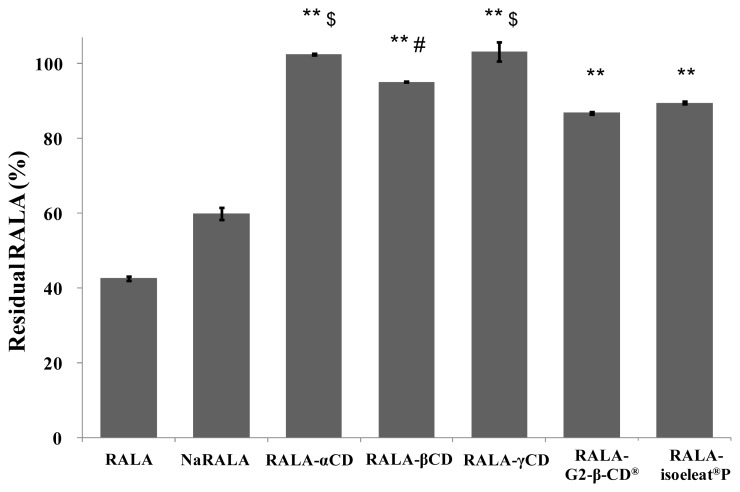
Stability of RALA complexes towards low pH. The samples were incubated at pH 1.2 and 37 °C for 1 h, and the remaining amount of RALA was quantified using HPLC. RALA in the RALA-CD complexes was significantly more stable than free RALA or NaRALA. ^**^p < 0.01 *vs.* RALA and NaRALA. $ *p* < 0.01 *vs.* RALA-βCD, RALA-G2-β-CD^®^ and RALA-isoeleat^®^P. # *p* < 0.01 *vs.* RALA-G2-β-CD^®^ and RALA-isoeleat^®^P. (Fisher’s least significant difference test)

Figure 4 shows the remaining RALA percentages of free RALA, NaRALA and the RALA-CD complexes after incubation with the stomach acid imitation. While free RALA was very unstable (43% of the incubated RALA remained) and NaRALA was only slightly more stable (60% of the incubated RALA resisted degradation) than RALA, complex formation with any of the tested cyclodextrins improved the RALA yield significantly. However, RALA-αCD and RALA-γCD showed the highest stabilization of the incorporated RALA, which was almost 100%. The differences between RALA-αCD, RALA-βCD and RALA-γCD compared with RALA-G2-β-CD^®^ and RALA-isoeleat^®^P were statistically significant, whereby RALA-αCD and RALA-γCD were also more stable than RALA-βCD (statistically significant). Additionally, these results indicate that G2-β-CD^®^, maltosyl-β-CD and isoeleat^®^P (a mixture of α-, β- and γ-CDs, maltosyl-α-, -β- and -γ-CDs) are water-soluble cyclodextrins, and their decreased stability under acidic conditions could be due to being dissolved as opposed to being in suspension.

Interestingly, the imitated stomach acid samples had a different physical appearance depending on the RALA formulation they contained. While non-complexed RALA polymerizes in the acidic environment, the RALA-γCD complex remains suspended and no signs of polymerization are observed. Similar effects are seen for the other RALA-CD complexes (data not shown).

In these experiments, we have been able to show that complex formation with cyclodextrins, and in particular with α-CD and γ-CD, stabilizes RALA under acidic conditions. Similar to what we have demonstrated regarding the NaRALA heat and humidity stability that is inferior to RALA-CD complexes, RALA in RALA-CD complexes are significantly more stable towards low pH than the sodium salt.

### 2.5. Scanning Electron Microscopy of the Different RALA-CD Complexes

Having found differences regarding RALA stability in the studied RALA-cyclodextrin complexes, we also analyzed the different complexes using SEM. These experiments showed that the shape and aspect of the complex particles differed considerably and depended on the cyclodextrin used for complex formation.

**Figure 5:**
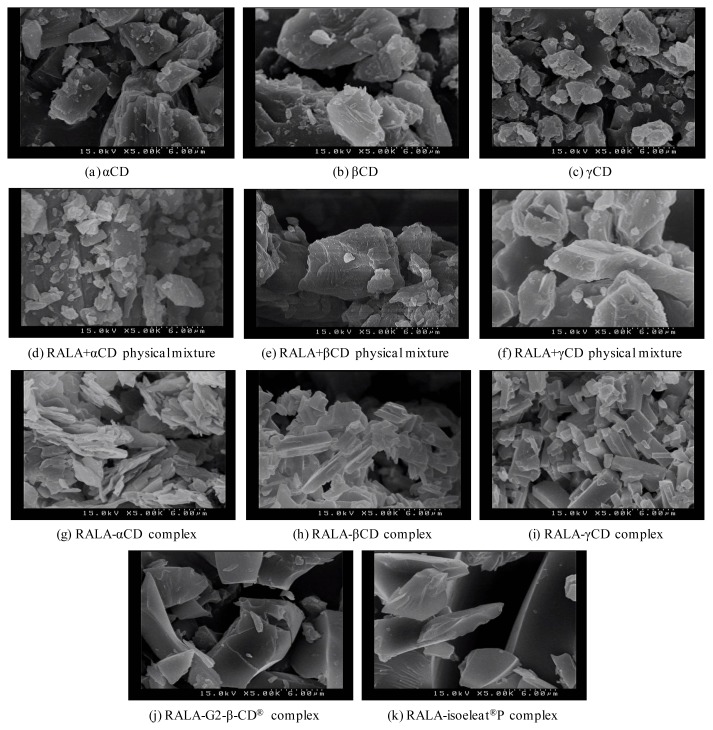
SEM images of CD, RALA + CD physical mixtures and RALA-CD complexes. (**a**) αCD; (**b**) βCD; (**c**) γCD; (**d**) RALA + αCD physical mixture; (**e**) RALA + βCD physical mixture; (**f**) RALA + γCD physical mixture; (**g**) RALA-αCD complex; (**h**) RALA-βCD complex; (**i**) RALA-γCD complex; (**j**) RALA-G2-β-CD^®^ complex; (**k**) RALA-isoeleat^®^P complex.

Figure 5 shows SEM images of CDs, RALA + CD physical mixtures and RALA-CD complexes. CDs and RALA + CD physical mixtures contain particles that appear cracked and wrinkled, their shapes are uneven and particle sizes vary considerably in the physical mixtures, with the maximum RALA + CD physical mixture particle size exceeding 50 μm. There was no considerable change observed between the CD and its physical mixture with RALA in SEM analysis.

This observation was confirmed in three different fields within each sample of which images with 300-, 500-, 1000- and 5000-times magnification were taken. Figure 5 shows the highest magnification (5000). Contrarily, the particle size distribution of RALA-αCD, RALA-βCD and RALA-γCD complexes appears more homogenous than in the RALA + CD physical mixtures. Particles of RALA-CD complexes form larger aggregates, which seem to pile up.

Additionally, the RALA-βCD and RALA-γCD complex crystals are shaped like prisms with parallel sides, and in the case of the rod-shaped RALA-γCD particles, there are tetragonal and orthorhombic crystals to be seen in the SEM images. While the RALA-βCD and RALA-γCD complex particles have rather smooth surfaces, the particles that constitute the RALA-αCD complex are rougher, and the shapes formed by these crystals seldom show square and parallel surfaces. These results show that the morphological characteristics of RALA-CD complexes are different from CDs or RALA + CD physical mixtures, and it appears that RALA-CD complex particles form crystals during their manufacturing process.

The RALA complexes with G2-β-CD^®^ and isoeleat^®^P form particles with smooth and flat surfaces, although their shapes are not symmetrical, nor do they have parallel surfaces. The particle sizes are also much bigger for RALA-G2-β-CD^®^ and RALA-isoeleat^®^P complexes than for RALA-αCD, RALA-βCD and RALA-γCD complexes (Figure 5).

**Figure 6:**
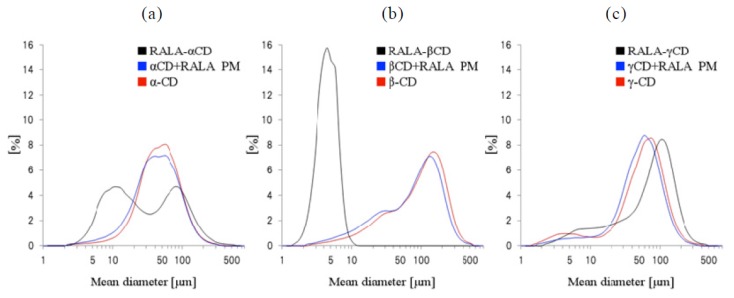
Particle size distributions of the RALA-αCD, -βCD and -γCD complexes, their physical mixtures (PM) and CD. (**a**) α-CD, physical mixture and RALA-αCD, (**b**) β-CD, physical mixture and RALA-βCD and (**c**) γ-CD, physical mixture and RALA-γCD.

To further examine the particle morphology, we conducted a particle size distribution analysis of the CDs, physical mixtures and RALA-CD complexes using a particle size distribution analyzer (Figure 6). In this measurement, α-CD showed a single peak, and the mean median diameter was 43 μm. The RALA + αCD physical mixture also showed only one peak, and the mean median diameter was 40 μm. Contrarily, RALA-αCD showed two peaks, one at a smaller diameter (13 μm) and one at a larger diameter (77 μm) than the peak of α-CD.

RALA-βCD showed one sharp peak at a smaller diameter than the peak of β-CD itself. On complexing RALA, the particle size peak for β-CD shifted from 152 μm to 6 μm for the RALA-βCD complex, thereby differing considerably from the peaks and type of peak shifts observed for complexation with α-CD. The RALA + γCD physical mixture showed one peak with an average mean diameter of 52 μm. This average mean diameter is almost the same as for γ-CD on its own (55 μm). The mean median diameter of the RALA-γCD complex was 76 μm. In the case of γ-CD, there did not seem to be great differences between the cyclodextrin on its own, the physical mixture or the complex concerning the particle size distribution pattern, although the SEM images showed considerable morphology changes upon physical mixing and complexation.

In order to confirm the difference of the particle size distributions of RALA-γCD and γ-CD or RALA + γCD physical mixture, we carried out an *F*-test and *t*-test. The F-test results revealed that the particle size distributions of RALA-γCD and γ-CD or the physical mixture were not significantly different. Next, a *t*-test clarified that the mean particle size of RALA-γCD and γ-CD or the physical mixture were completely different. The SEM images of RALA-γCD showed a characteristic rod shape, which was not observed in the cases of γ-CD or the RALA + γCD physical mixture. These results can be explained by considering the difference of the mean particle size of RALA-γCD, RALA + γCD physical mixture and γ-CD itself.

The particle size distribution measurement showed different results for RALA-γCD compared to RALA-αCD and RALA-βCD. On the one hand, in the case of the αCD- and βCD-complexes, the particle size distributions changed when the cyclodextrin complexed RALA. This change is consistent with differences observed between the complexes and the physical mixtures in the SEM images. On the other hand, in the case of γ-CD, the particle size distributions seem to remain unchanged upon complexation, although differences between the mean particle size and the SEM images can be clearly observed. These results suggest that the manufacturing process affected the particle size distributions of RALA-αCD and RALA-βCD, but did not affect that of RALA-γCD. Nevertheless, the manufacturing process influenced the particle shapes for all CD complexes and affected the stability in the DSC analysis.

**Figure 7:**
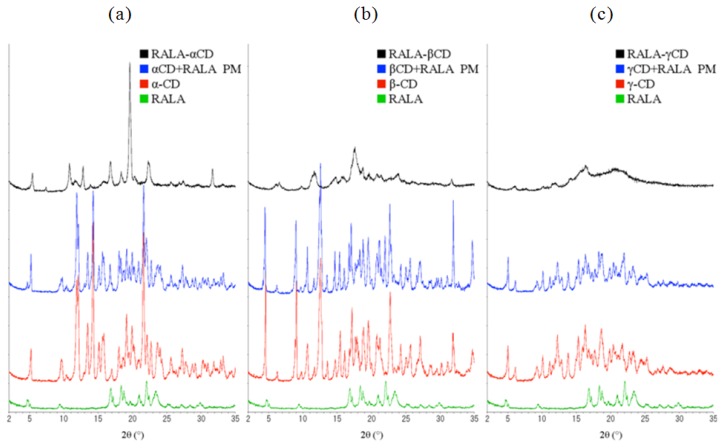
XRD patterns of the RALA-αCD, -βCD and -γCD complexes, their physical mixtures (PM), CD and free RALA. (**a**) α-CD, physical mixture, RALA-αCD and free RALA, (**b**) β-CD, physical mixture, RALA-βCD and free RALA and (**c**) γ-CD, physical mixture, RALA-γCD and free RALA.

Figure 7 shows the X-ray diffraction patterns of α-, β- and γ-CD compared with free RALA, the physical mixture of RALA with the different CDs and the RALA-CD complexes.

As can be seen in Figure 7, there are four characteristic diffraction peaks for RALA at 2θ = 17, 18.5, 21 and 22. α-, β- and γ-CD differed from each other in their diffraction patterns, and the physical mixture diffraction patterns appeared as the addition of CD and RALA patterns.

Contrarily, complexation of RALA with the CDs changed the XRD patterns when compared to the physical mixtures. The RALA-αCD complex showed one large peak at 2θ = 19.6 (*d* = 4.53), which was not observed in the physical mixture. RALA-βCD showed fewer and smaller peaks than its physical mixture. RALA-γCD showed a broad diffraction profile, and the peaks observed for free RALA, CD or its physical mixture was absent.

The DSC, SEM studies and XRD analysis have shown that RALA forms complexes with all tested cyclodextrins and that these complexes have different physicochemical properties.

Cyclodextrins have been used for complex formation of various bioactive or pharmacologically active compounds, such as drugs and vitamin-like substances, thereby enhancing the bioavailability of these molecules [17–21]. Some of these studies also found that the physicochemical properties depended on the method used for complex formation [20]. In further experiments, it would be interesting to investigate whether different complex morphologies lead to different bioavailabilities *in vivo*.

## 3. Experimental Section

### 3.1. Chemicals

R(+)-alpha lipoic acid sodium salt (NaRALA) was purchased from ToyoHakko Co. Ltd. (Aichi, Japan). CAVAMAX^®^ W6 FOOD (α-CD), CAVAMAX^®^ W7 FOOD (β-CD) and CAVAMAX^®^ W8 FOOD (γ-CD) were purchased from Wacker Chemie AG (München, Germany). G2-β-CD^®^ (maltosyl-β-CD) and Isoeleat^®^P (a commercially available product containing α-, β- and γ-CDs, maltosyl-α-, -β- and -γ-CDs, as well as maltose and glucose) were supplied by Ensuiko Sugar Refining (Tokyo, Japan). The solvents used for spectrophotometry were purchased from Wako Pure Chemical Ind., Ltd. (Osaka, Japan). All reagents used were analytical grade, and Milli Q^®^ water was used throughout the study.

### 3.2. Equipment

The mechanical stirrer (EYELA NZ-1000) and the freeze dryer (EYELA FD-1000) used in this study were from Tokyo Rika Kikai, Japan.

Differential scanning calorimetry (DSC) was carried out using the DSC-60 thermal analyzer (Shimadzu, Kyoto, Japan). The high pressure liquid chromatography (HPLC) system LC-2010 was equipped with a high-speed autosampler, a UV-Vis detector and the LC solution^®^ chromatography data system software (Shimadzu, Kyoto, Japan).

The scanning electron microscope (SEM) used was the S-4500 from HITACHI, Japan.

The particle size distribution analysis was carried out using a LA-920 system (Horiba, Japan).

Powder X-ray diffraction patterns (XRD) were measured with a Rint 2200 diffractometer (Rigaku, Tokyo, Japan).

### 3.3. Preparation of the RALA-CD Complexes

5 g of NaRALA were dissolved in 120 mL water, and the corresponding molar amount of α-CD (25 g), β-CD (31 g), γ-CD (33 g), G2-β-CD^®^ (36 g) or isoeleat^®^P (42 g) for a 1:1 ratio with NaRALA was added. The solution was mixed with a mechanical stirrer at 300 rpm for 10 min before adding 24 mL 1 M HCl dropwise over a time period of 30 min. Then, the suspension was continuously stirred in the dark for 18 h. All procedures were carried out at room temperature, and the suspension temperature did not exceed 25 °C. The freshly prepared suspension was frozen overnight and freeze dried the next day.

For the physical mixtures, RALA and the corresponding molar amounts of the different cyclodextrins for a 1:1 ratio were mixed in a mortar.

### 3.4. Differential Scanning Calorimetry (DSC)

For the differential scanning calorimetry (DSC), 5 mg of the analyzed RALA-CD complexes were placed in sealed aluminum pans under air flow for scanning and heating from 30 °C to 100 °C at a heating rate of 3 °C/min.

### 3.5. Measurement of RALA Content in the RALA-CD Complexes Using HPLC

The percentage of RALA contained in the RALA-CD complexes was analyzed by HPLC using a chiral column (CHIRALPAK AD-RH Daicel; 4.6 mm I.D. × 150 mm) with a mobile phase consisting of 5 mM H_3_PO_4_/acetonitrile (70:30, *v*/*v*) at a flow rate of 0.6 mL/min and at a temperature of 25 °C. Lipoic acid was detected at 215 nm, based on the current literature [22], and the injection volume was 10 μL. Racemic dl-alpha lipoic acid (Fluka, Newport News, VA, USA) was used as a standard, the stock solution was prepared at 0.5 mg/mL in 25 mM KH_2_PO_4_ buffer (pH 3.5)/acetonitrile (50:50, *v*/*v*) and filtered (ADVANTEC DISMIC-25, 0.2 μm). In this solvent, the RALA-CD complexes dissociated, and the free RALA could be detected. By using a chiral column, it was possible to separate the enantiomers; therefore, obtaining different peaks for RALA and SALA. Retention time of SALA was 34–35 min and that of RALA 36–38 min. The sample content was calculated by using the peak area. Sample preparations were conducted 3 times for each sample, and the calculated relative standard deviation (RSD) was smaller than 3%.

### 3.6. Thermal Stability of the RALA-CD Complexes Using HPLC

20 mg RALA-γCD complex or 5 mg of free RALA (Changshu Fushilai) powder were incubated in an open amber glass bottle for 30 min, 1 h, 2 h, 5 h, 24 h and 48 h at 25 °C or 70 °C under 100% relative humidity conditions (imitating unfavorable storage conditions). Subsequently, 25 mM KH_2_PO_4_ buffer (pH 3.5)/acetonitrile (50:50, *v*/*v*) was added to the glass bottle before mixing and sonification of the sample. After filtration (ADVANTEC DISMIC, 0.2 μm), the sample was analyzed using HPLC, as described above. The thermal stability of free RALA and RALA in the RALA-γCD complex was expressed as the percentage of recovered RALA after the heat incubation.

Sample preparations were conducted 3 times for each sample, and the calculated RSD was smaller than 3%.

### 3.7. Stability under Acidic Conditions

We evaluated the stability of the RALA-CD complexes under acidic conditions using a gastric fluid model (0.2% NaCl in water, pH adjusted to 1.2 with HCl).

170 mg of each RALA-CD complex were put into an amber glass bottle, and 5 mL of the simulated gastric fluid were added. The bottle was sealed and kept in a water bath at 37 °C for 60 min (imitating the passage through the stomach). After the treatment, 50 μL sample suspension were taken and diluted with 950 μL 25 mM KH_2_PO_4_ buffer (pH 3.5)/acetonitrile (50:50, *v*/*v*). Then, the sample solution was filtered (ADVANTEC DISMIC, 0.2 μm), and the amount of RALA was assayed using HPLC. System conditions were the same as described above. The stability of RALA in the RALA-CD complex and free RALA under acidic conditions was expressed as the percentage of recovered RALA after low pH incubation.

Sample preparations were conducted 3 times for each sample, and the calculated RSD was smaller than 3%.

### 3.8. Morphological Characterization via Scanning Electron Microscopy (SEM) Analysis

For scanning electron microscopy analysis, the RALA-CD complexes were sprinkled onto conductive glue on a palladium SEM stub and sputter coated with gold for 3 min. Then, the RALA-CD complexes were measured at 15 kV with the SEM S-4500, HITACHI, for morphology analysis. Three different fields within each sample were randomly chosen, and 4 images of each field were taken at the magnifications 300, 500, 1000 and 5000, giving a total number of 12 images per sample.

### 3.9. Particle Size Distribution

The solid CD was gradually added to 150 mL continuously stirred ethanol in the particle size distribution analyzer until reaching the adequate measurement range (lower than 95% transmittance). The measurement was carried out at a refractive index of 1.30. After each measurement, the ethanol was discharged. In order to clean the container, it was filled with water and sonificated for 1 minute. Then, the container was emptied and filled with fresh ethanol for the following sample.

Measurements were carried out 3 times for each sample.

### 3.10. X-Ray Diffraction (XRD) Measurements

XRD measurements were performed using a powder diffractometer (Rigaku, Rint 2200, Tokyo, Japan) with monochromated Cu-Kα radiation. The acceleration voltage and current were 40 kV and 40 mA, respectively. The solid α-, β- or γ-CD, the RALA, the physical mixtures of the different CDs with RALA or the RALA-CD complexes were put on the glass plates. The θ/2θ continuous scans were performed in the 2θ range of 2°–35° with a scanning speed of 1 °/min.

### 3.11. Statistical Analyses

Data are expressed as means ± SD. The significance of difference between groups was assessed by analysis of variance (ANOVA) with a Fisher’s least significant difference test. Differences were considered significant when the *p* value was ≤0.05.

## 4. Conclusions

RALA is known to be a powerful antioxidant and plays an important role in metabolism [4]. Although some scientific evidence, such as long-term intervention studies, is still missing, pharmacological use of RALA, as well as its use as a food supplement, seems beneficial for human health [23]. Therefore, it is of great interest to improve RALA stability in order to facilitate its storage, application and ingestion. Also, stabilization of the ALA isomers makes it possible to study the bioactive properties of RALA and SALA separately, which is especially relevant, as the commercially available α-lipoic acid is racemic, while the naturally occurring molecule is the enantiomer RALA.

From our data, we conclude that γ-CD is the best suited cyclodextrin for RALA complex formation (see Table 1). The DSC scan does not show a peak that would otherwise indicate instability, very little RALA is lost during the preparation of the complex and, in our tests, the RALA-γCD complex was the most stable towards heat, humidity and low pH, as was shown via quantification of the residual RALA by HPLC. The morphology of RALA-CD mixtures changes upon complex formation and differs depending on the CD used for the complex formation. The RALA-γCD complex formed the most distinctive type of crystals, as these particles were rod-shaped.

Further studies, including *in vivo* experiments, are needed to explore the bioavailability and biological activity of the RALA-CD complexes in order to evaluate their potential use as nutraceuticals, pharmaceuticals and cosmeceuticals. We are currently studying the absorption mechanism of orally administrated RALA-CD in rats. McCormick and co-workers investigated the metabolism of dl-[1,6-^14^C] lipoic acid in rats and found that most of the racemate is metabolized via β-oxidation of the valeric acid side chain [24]. Having found a way to feed enantiopure RALA, it would also be interesting to investigate whether this or its administration as a complex affects its metabolism.

However, considering recent data [8,13], including this study, the potential use of cyclodextrin-complexed RALA in foods, drugs and pharmaceuticals seems very promising.

## Figures and Tables

**Table 1:** Summarized stability test results of the analyzed RALA-CD complexes.

(Sample)	DSC Figure 1	Yield in manufacturing process Figure 2	Thermal stability (70 °C, 48 h) Figure 3b	Acidic stability Figure 4
RALA-αCD	No peak	75% ± 0.39% *	98% ± 1.25% *	~100% **
RALA-βCD	No peak	97% ± 1.24% *	69% ± 1.83% *	95% ± 0.14% *
RALA-γCD	No peak	97% ± 0.87% *	~100% **	~100% **
RALA-G2-β-CD^®^	No peak	70% ± 0.13% *	~100% **	87% ± 0.26% *
RALA-isoeleat^®^P	Peak shift	90% ± 0.83% *	69% ± 0.27% *	90% ± 0.36% *

* mean ± S.D;

** Almost complete recovery.

## References

[1] Packer L., Cadenas E. (2011). Lipoic acid: Energy metabolism and redox regulation of transcription and cell signaling. J. Clin. Biochem. Nutr.

[2] Smith A.R., Shenvi S.V., Widlanski M., Suh J.H., Hagen T.M. (2004). Lipoic acid as a potential therapy for chronic diseases associated with oxidative stress. Curr. Med. Chem.

[3] Matsugo S., Yan L.J., Konishi T., Youn H.D., Lodge J.K., Ulrich H., Packer L. (1997). The lipoic acid analogue 1,2-diselenolane-3-pentanoic acid protects human low density lipoprotein against oxidative modification mediated by copper ion. Biochem. Biophys. Res. Commun.

[4] Packer L., Kraemer K., Rimbach G. (2001). Molecular aspects of lipoic acid in the prevention of diabetes complications. Nutrition.

[5] Hagen T.M., Ingersoll R.T., Lykkesfeldt J., Liu J., Wehr C.M., Vinarsky V., Bartholomew J.C., Ames B.N. (1999). (R)-α-lipoic acid-supplemented old rats have improved mitochondrial function, decreased oxidative damage, and increased metabolic rate. FASEB J..

[6] Tirosh O., Sen C.K., Roy S., Kobayashi M.S., Packer L. (1999). Neuroprotective effects of α-lipoic acid and its positively charged amide analogue. Free Radic. Biol. Med.

[7] Matsugo S., Han D., Tritschler H.J., Packer L. (1996). Decomposition of alpha-lipoic acid derivatives by photoirradiation-formation of dihydrolipoic acid from alpha-lipoic acid. Biochem. Mol. Biol. Int.

[8] Kofuji K., Nakamura M., Isobe T., Murata Y., Kawashima S. (2008). Stabilization of á-lipoic acid by complex formation with chitosan. Food Chem.

[9] Weerakody R., Fagan P., Kosaraju S.L. (2008). Chitosan microspheres for encapsulation of alpha-lipoic acid. Int. J. Pharm.

[10] Carlson D.A., Smith A.R., Fischer S.J., Young K.L., Packer L. (2007). The plasma pharmacokinetics of R-(+)-lipoic acid administered as sodium R-(+)-lipoate to healthy human subjects. Altern. Med. Rev.

[11] Gao X., Nishimura K., Hirayama F., Arima H., Uekama K., Schmid G., Terao K. (2006). Enhanced dissolution and oral bioavailability of coenzyme Q10 in dogs obtained by inclusion complexation with γ-cyclodextrin. Asian J. Pharm. Sci.

[12] Yadav V.R., Suresh S., Devi K., Yadav S. (2009). Effect of cyclodextrin complexation of curcumin on its solubility and antiangiogenic and anti-inflammatory activity in rat colitis model. AAPS Pharm. Sci. Tech.

[13] Takahashi H., Bungo Y., Mikuni K. (2011). The aqueous solubility and thermal stability of α-lipoic acid are enhanced by cyclodextrin. Biosci. Biotechnol. Biochem.

[14] Hoppe P.P., Krennrich G. (2000). Bioavailability and potency of natural-source and all-racemic α-tocopherol in the human: A dispute. Eur. J. Nutr.

[15] Jensen S.K., Lauridsen C. (2007). Alpha-tocopherol stereoisomers. Vitam. Horm.

[16] Muller P.Y., Netscher T., Frank J., Stoecklin E., Rimbach G., Barella L. (2005). Comparative quantification of pharmacodynamic parameters of chiral compounds (RRR- *vs*. all-rac-α tocopherol) by global gene expression profiling. J. Plant Physiol.

[17] Baek H.H., Kwon S.Y., Rho S.J., Lee W.S., Yang H.J., Hah J.M., Choi H.G., Kim Y.R., Yong C.S. (2011). Enhanced solubility and bioavailability of flurbiprofen by cycloamylose. Arch. Pharm. Res.

[18] De Jesus M.B., de Matos Alves Pinto L., Fraceto L.F., Magalhaes L.A., Zanotti-Magalhaes E.M., de Paula E. (2010). Improvement of the oral praziquantel anthelmintic effect by cyclodextrin complexation. J. Drug Target.

[19] Hatanaka J., Kimura Y., Lai-Fu Z., Onoue S., Yamada S. (2008). Physicochemical and pharmacokinetic characterization of water-soluble Coenzyme Q10 formulations. Int. J. Pharm.

[20] Pires M.A., Souza Dos Santos R.A., Sinisterra R.D. (2011). Pharmaceutical composition of hydrochlorothiazide:β-cyclo-dextrin: Preparation by three different methods, physico-chemical characterization and *in vivo* diuretic activity evaluation. Molecules.

[21] Yallapu M.M., Jaggi M., Chauhan S.C. (2010). β-Cyclodextrin-curcumin self-assembly enhances curcumin delivery in prostate cancer cells. Colloid Surf. B.

[22] Durrani A.I., Schwarz H., Schmid W., Sontag G. (2007). α-Lipoic acid in dietary supplements: Development and comparison of HPLC-CEAD and HPLC-ESI-MS methods. J. Pharm. Biomed. Anal.

[23] Goraca A., Huk-Kolega H., Piechota A., Kleniewska P., Ciejka E., Skibska B. (2011). Lipoic acid—Biological activity and therapeutic potential. Pharmacol. Rep.

[24] McCormick D.B., Harrison E.H. (1974). The metabolism of dl-[1,6-^14^C] lipoic acid in the rat. Arch. Biochem. Biophys.

